# Supplementary material for: The transcriptomic and epigenetic map of vascular quiescence in the continuous lung endothelium
Source: eLife. 2018 May 11;7:e34423. doi: 10.7554/eLife.34423 (PMC5947988; doi:10.7554/eLife.34423)
Supplement: Figure 1—source data 1. — For each pathway the adjusted p‐value and the number of proteins, that is, count, are specified. [file elife-34423-fig1-data1.docx]

**Figure 1 – source data 1.** Functionally enriched KEGG pathways Identified for (i) seeds in the EC maturation network, (ii) proteins in the EC maturation network as well as (iii) for all 3,223 seeds. For each pathway the adjusted p‐value and the number of proteins, i.e., count, are specified.

|  |  | Spoke SEEDS UP&DW | | DIAMOnD network UP&DW | | All SEEDS UP&DW | |
| --- | --- | --- | --- | --- | --- | --- | --- |
| KEGG pathways | KEGG IDs | p-value | count | p-value | count | p-value | count |
| Jak-STAT signaling pathway | KEGG:04630 |  |  | 5.72E-11 | 39 |  |  |
| Osteoclast differentiation | KEGG:04380 |  |  | 6.23E-09 | 35 |  |  |
| Axon guidance | KEGG:04360 |  |  | 3.55E-07 | 39 |  |  |
| Toxoplasmosis | KEGG:05145 |  |  | 2.24E-06 | 30 | 0.00071 | 29 |
| Prolactin signaling pathway | KEGG:04917 |  |  | 2.92E-06 | 23 |  |  |
| T cell receptor signaling pathway | KEGG:04660 |  |  | 3.01E-06 | 29 |  |  |
| Fc epsilon RI signaling pathway | KEGG:04664 |  |  | 4.19E-06 | 21 |  |  |
| AGE-RAGE signaling pathway in diabetic complications | KEGG:04933 | 0.00045 | 26 | 9.47E-06 | 27 | 1.14E-07 | 34 |
| Focal adhesion | KEGG:04510 |  |  | 1.02E-05 | 38 | 0.00168 | 43 |
| Natural killer-mediated cytotoxicity | KEGG:04650 |  |  | 1.26E-05 | 25 |  |  |
| Pathways in cancer | KEGG:05200 | 0.0327 | 56 | 1.71E-05 | 61 | 2.08E-07 | 83 |
| MicroRNAs in cancer | KEGG:05206 |  |  | 1.80E-05 | 32 | 0.0127 | 31 |
| B cell receptor signaling pathway | KEGG:04662 |  |  | 2.04E-05 | 22 |  |  |
| Glycolysis / gluconeogenesis | KEGG:00010 |  |  | 4.21E-05 | 15 |  |  |
| Neurotrophin signaling pathway | KEGG:04722 |  |  | 5.48E-05 | 30 |  |  |
| Proteoglycans in cancer | KEGG:05205 |  |  | 5.54E-05 | 40 |  |  |
| PI3K-Akt signaling pathway | KEGG:04151 |  |  | 5.60E-05 | 46 | 0.00029 | 66 |
| Hypertrophic cardiomyopathy | KEGG:05410 | 0.0244 | 16 | 5.70E-05 | 19 | 3.13E-05 | 27 |
| Rap1 signaling pathway | KEGG:04015 |  |  | 6.41E-05 | 36 |  |  |
| Dilated cardiomyopathy | KEGG:05414 | 0.00236 | 18 | 8.07E-05 | 19 | 7.58E-08 | 32 |
| Bacterial invasion of epithelial cells | KEGG:05100 |  |  | 9.47E-05 | 22 |  |  |
| Viral myocarditis | KEGG:05416 |  |  | 0.00014 | 16 | 3.26E-06 | 27 |
| Central carbon metabolism in cancer | KEGG:05230 |  |  | 0.00038 | 18 |  |  |
| Fc gamma R-mediated phagocytosis | KEGG:04666 |  |  | 0.00047 | 22 |  |  |
| ErbB signaling pathway | KEGG:04012 |  |  | 0.00061 | 22 |  |  |
| Adrenergic signaling in cardiomyocytes | KEGG:04261 | 0.00259 | 27 | 0.00075 | 26 | 0.00143 | 35 |
| cGMP-PKG signaling pathway | KEGG:04022 |  |  | 0.00170 | 27 | 0.00148 | 38 |
| Arrhythmogenic right ventri­cular cardiomyopathy (ARVC) | KEGG:05412 |  |  | 0.00216 | 15 | 0.00568 | 21 |
| HIF-1 signaling pathway | KEGG:04066 |  |  | 0.00256 | 23 |  |  |
| Measles | KEGG:05162 |  |  | 0.00343 | 26 |  |  |
| Platelet activation | KEGG:04611 |  |  | 0.00411 | 21 | 0.0355 | 27 |
| Primary immunodeficiency | KEGG:05340 |  |  | 0.00508 | 11 |  |  |
|  |  | Spoke SEEDS UP&DW | | DIAMOnD network UP&DW | | All SEEDS UP&DW | |
| KEGG pathways | KEGG IDs | p-value | count | p-value | count | p-value | count |
| Cell adhesion molecules | KEGG:04514 |  |  | 0.00620 | 21 | 9.83E-07 | 44 |
| Chronic myeloid leukemia | KEGG:05220 |  |  | 0.00810 | 19 |  |  |
| ECM-receptor interaction | KEGG:04512 |  |  | 0.00877 | 12 | 1.51E-10 | 34 |
| Circadian rhythm | KEGG:04710 |  |  | 0.01330 | 10 |  |  |
| Cardiac muscle contraction | KEGG:04260 | 0.0136 | 13 | 0.01680 | 12 |  |  |
| Chemokine signaling pathway | KEGG:04062 |  |  | 0.02090 | 28 |  |  |
| Alcoholism | KEGG:05034 |  |  | 0.02740 | 28 |  |  |
| Herpes simplex infection | KEGG:05168 |  |  | 0.02910 | 29 | 0.00482 | 42 |
| Hematopoietic cell lineage | KEGG:04640 |  |  | 0.02920 | 13 | 0.000413 | 25 |
| Type II diabetes mellitus | KEGG:04930 |  |  | 0.02920 | 13 |  |  |
| Phospholipase D signal. pathway | KEGG:04072 |  |  | 0.03200 | 21 |  |  |
| Hippo signaling pathway | KEGG:04390 |  |  | 0.03250 | 25 |  |  |
| Ras signalling pathway | KEGG:04014 |  |  | 0.03480 | 30 |  |  |
| Leukocyte transendoth migration | KEGG:04670 |  |  | 0.03770 | 21 |  |  |
| Metabolism of xenobiotics by cytochrome P450 | KEGG:00980 |  |  |  |  | 0.00713 | 19 |
| Drug metabolism-cytochrome P450 | KEGG:00982 |  |  |  |  | 0.000904 | 21 |
| Phagosome | KEGG:04145 |  |  |  |  | 0.00408 | 37 |
| Vascular smooth muscle contraction | KEGG:04270 |  |  |  |  | 0.0257 | 28 |
| Antigen processing and presentation | KEGG:04612 |  |  |  |  | 2.95E-07 | 29 |
| Regulation of lipolysis in adipocytes | KEGG:04923 |  |  |  |  | 0.00851 | 17 |
| Type I diabetes mellitus | KEGG:04940 |  |  |  |  | 0.000131 | 21 |
| Protein digestion and absorption | KEGG:04974 | 0.00033 | 14 |  |  | 0.000238 | 26 |
| Leishmaniasis | KEGG:05140 |  |  |  |  | 0.0488 | 17 |
| Malaria | KEGG:05144 |  |  |  |  | 1.75E-05 | 19 |
| Amoebiasis | KEGG:05146 |  |  |  |  | 0.0112 | 26 |
| HTLV-I infection | KEGG:05166 |  |  |  |  | 0.000447 | 56 |
| Small cell lung cancer | KEGG:05222 |  |  |  |  | 0.00237 | 23 |
| Autoimmune thyroid disease | KEGG:05320 |  |  |  |  | 0.0167 | 19 |
| Allograft rejection | KEGG:05330 |  |  |  |  | 0.000299 | 19 |
| Graft-versus-host disease | KEGG:05332 |  |  |  |  | 3.66E-05 | 21 |
