# Supplementary material for: The transcriptomic and epigenetic map of vascular quiescence in the continuous lung endothelium
Source: eLife. 2018 May 11;7:e34423. doi: 10.7554/eLife.34423 (PMC5947988; doi:10.7554/eLife.34423)
Supplement: Figure 1—source data 2. — The highlighted genes were selected for validation by qPCR (see Figure 1D and Figure 1 – figure supplement 1E). [file elife-34423-fig1-data2.docx]

Figure 1 – source data 2. Cell cycle genes regulated in EC during the transition to a quiescent state. The highlighted genes were selected for validation by qPCR (see Figure 1D and Figure 1 – figure supplement 1E).

| **Cell cycle**  **gene** | **log2FC**  **yAdu vs. inf** | **adj.**  **p-value** |
| --- | --- | --- |
| ***Mis18bp1*** | -5.07 | 1.79E-07 |
| ***Birc5*** | -4.68 | 7.85E-12 |
| ***Ccnb1*** | -4.56 | 2.29E-06 |
| ***Ccnb2*** | -4.55 | 2.67E-18 |
| ***Aurkb*** | -4.22 | 8.93E-05 |
| ***Cdk1*** | -3.94 | 9.22E-13 |
| ***Kif23*** | -3.85 | 3.09E-18 |
| ***Bub1*** | -3.71 | 1.26E-03 |
| ***Casc5*** | -3.51 | 1.23E-10 |
| ***Nuf2*** | -3.47 | 9.61E-04 |
| ***Ube2c*** | -3.43 | 6.58E-08 |
| ***Rrm2*** | -3.36 | 1.08E-18 |
| ***Spc24*** | -3.33 | 1.77E-05 |
| ***Cenpm*** | -3.27 | 1.99E-05 |
| ***Kif2c*** | -3.08 | 1.31E-02 |
| ***Cenpk*** | -2.93 | 2.13E-06 |
| ***Plk1*** | -2.91 | 9.68E-09 |
| ***Cenph*** | -2.8 | 4.99E-04 |
| ***Cdca8*** | -2.79 | 7.76E-08 |
| ***Bub1b*** | -2.68 | 9.58E-04 |
| ***Cenpi*** | -2.37 | 5.09E-05 |
| ***Ninl*** | -2.26 | 7.83E-06 |
| ***Cks1b*** | -2.1 | 2.14E-07 |
| ***Cdkn2c*** | -2.08 | 5.90E-03 |
| ***E2f2*** | -2.06 | 1.59E-02 |
| ***Ndc80*** | -1.9 | 2.05E-03 |
| ***Pole*** | -1.84 | 1.44E-03 |
| ***Hist1h2ae*** | -1.78 | 3.02E-02 |
| ***Zwilch*** | -1.67 | 3.12E-03 |
| ***Prkar2b*** | -1.57 | 1.38E-02 |
| ***Cdc7*** | -1.5 | 1.47E-09 |
| ***Tubg2*** | -1.44 | 4.38E-04 |
| ***E2f3*** | -1.44 | 5.91E-04 |
| ***Myc*** | -1.38 | 7.10E-08 |
| ***Cenpa*** | -1.33 | 2.26E-04 |
| ***Fbxo5*** | -1.31 | 2.88E-03 |
| ***Gins2*** | -1.29 | 2.01E-03 |
| ***Cdc20*** | -1.28 | 1.28E-02 |
| ***Cenpp*** | -1.26 | 6.04E-03 |
| ***Aurka*** | -1.25 | 2.99E-03 |
| ***Cenpq*** | -1.24 | 1.03E-04 |
| ***Spc25*** | -1.21 | 2.38E-04 |
| ***Nsl1*** | -1.19 | 3.09E-02 |
| ***Kif20a*** | -1.15 | 9.03E-04 |
| ***Dbf4*** | -1.14 | 7.79E-04 |
| ***Incenp*** | -1.09 | 4.11E-03 |
| ***Wee1*** | -1.03 | 1.77E-02 |
| ***H2afx*** | -1.02 | 9.07E-04 |
| ***Ccnd2*** | -0.99 | 3.95E-04 |
| ***Pmf1*** | -0.87 | 8.21E-04 |
| ***Lmna*** | -0.87 | 1.18E-03 |
| ***Lin9*** | -0.87 | 1.06E-02 |
