# Supplementary material for: The transcriptomic and epigenetic map of vascular quiescence in the continuous lung endothelium
Source: eLife. 2018 May 11;7:e34423. doi: 10.7554/eLife.34423 (PMC5947988; doi:10.7554/eLife.34423)
Supplement: Supplementary file 1. [file elife-34423-supp1.docx]

*Key resource table*

| **Reagent type (species) or resource** | **Designation** | **Source or reference** | **Identifiers** | **Additional information** |
| --- | --- | --- | --- | --- |
| gene (*Mus musculus*) | Smad6 | N/A | MGI:1336883; NCBI Gene: 17130 |  |
| gene (*Mus musculus*) | Smad7 | N/A | MGI:1100518; NCBI Gene: 17131 |  |
| gene (*Homo sapiens*) | SMAD6 | N/A | HGNC:6772; NCBI Gene: 4091 |  |
| gene (*Homo sapiens*) | SMAD7 | N/A | HGNC:6773; NCBI Gene: 4092 |  |
| genetic reagent (*Mus musculus*) | pGIPZ-ns-shRNA | Dharmacon | Cat#RHS4348 |  |
| genetic reagent (*Mus musculus*) | pGIPZ-shSMAD6-118 | Dharmacon | Cat#V3LHS_340634 |  |
| genetic reagent (*Mus musculus*) | pGIPZ-shSMAD7-115 | Dharmacon | Cat#V3LHS_304614 |  |
| strain, strain background (*Mus musculus*) | C57BL/6N | Taconic Biosciences | C57BL/6NTac |  |
| primary cells (*Homo sapiens*) | HUVEC (human umbilical vein endothelial cells) | Promocell | Cat#C-12203 | primary human endothelial cells |
| biological sample (*Mus musculus*) | infant mouse endothelial cells | This paper | N/A | isolated by FACS from infant mice (8-10 days of age) |
| biological sample (*Mus musculus*) | young adult mouse endothelial cells | This paper | N/A | isolated by FACS from young adult mice (8-12 weeks of age) |
| antibody | ACTB (rabbit-anti-mouse/human) | Santa Cruz Biotechnol. | Cat#sc-1616, RRID:AB_630836 | 1:5000 for immunoblot |
| antibody | CD31 (rat-anti-mouse, APC-labelled) | BD Biosciences | Cat#551262, RRID:AB_398497 | 1:100 for FACS |
| antibody | CD31 (rat-anti-mouse) | BD Biosciences | Cat#553370,  RRID:AB_394816 | 1:50 for IF staining |
| antibody | CD34 (rat-anti-mouse, Pacific Blue-labelled) | eBioscience | Cat#48-0341, RRID:AB_2043838 | 1:50 for FACS |
| antibody | IgG (goat-anti-rabbit, Alexa546-labelled) | ThermoFisher Scientific | Cat#A11071, RRID:AB_1500774 | 1:500 for IF staining |
| antibody | IgG (goat-anti-rabbit, HRP-labelled) | Dako | Cat#P0448, RRID:AB_2617138 | 1:5000 for immunoblot |
| antibody | IgG (goat-anti-rat, Alexa488-labelled) | ThermoFisher Scientific | Cat#A11006, RRID:AB_141373 | 1:500 for IF staining |
| antibody | KI67 (rabbit-anti-mouse) | Gene Tex | Cat#GTX 16667, RRID:AB_422351 | 1:100 for IF staining |
| antibody | LY76 (rat-anti-mouse, FITC-labelled) | BD Biosciences | Cat#561032, RRID:AB_10563083 | 1:200 for FACS |
| antibody | LYVE1 (rat-anti-mouse, FITC-labelled) | eBioscience | Cat#53-0443, RRID:AB_1633415 | 1:250 for FACS |
| antibody | PDPN (hamster-anti-mouse, Alexa488-labelled) | eBioscience | Cat#53-5381, RRID:AB_1106991 | 1:100 for FACS |
| antibody | pSMAD1/5/8 (rabbit-anti-mouse/human) | Maine Medical Center | Cat#D6656/Vli31 | 1:2000 for immunoblot |
| antibody | pSMAD2/3 (rabbit-anti-mouse/human) | Maine Medical Center | Cat#D6658 | 1:2000 for immunoblot |
| antibody | PTPRC (rat-anti-mouse, FITC-labelled) | BD Biosciences | Cat#553080, RRID:AB_394610 | 1:400 for FACS |
| antibody | SMAD1 (rabbit-anti-mouse/human) | Cell Signaling | Cat#9743S, RRID:AB_2107780 | 1:1000 for immunoblot |
| antibody | SMAD2/3 (rabbit-anti-mouse/human) | Cell Signaling | Cat#5678 | 1:1000 for immunoblot |
| recombinant DNA reagent | pLenti-CMV-EV-neomycin^Res^ | Addgene, RRID: SCR_002037 | Cat#17392 |  |
| recombinant DNA reagent | pLenti-CMV-EV-puromycin^Res^ | Addgene, RRID: SCR_002037 | Cat#17452 |  |
| recombinant DNA reagent | pLenti-CMV-SMAD6-puromycin^Res^ | This paper | N/A | progenitor: pLenti CMV Puro DEST (w118-1) (Addgene, 17452), insert: NM_005585.4 |
| recombinant DNA reagent | pLenti-CMV-SMAD7-neomycin^Res^ | This paper | N/A | progenitor: pLenti CMV Neo DEST (705-1) (Addgene, 17392), insert: NM_005904.3 |
| sequence-based reagent | Acta2 | ThermoFisher Scientific | Mm00725412_s1 |  |
| sequence-based reagent | Actb | ThermoFisher Scientific | Mm00607939_s1 |  |
| sequence-based reagent | Bmpr2 | ThermoFisher Scientific | Mm00432134_m1 |  |
| sequence-based reagent | Ccnb1 | ThermoFisher Scientific | Mm03053893_gH |  |
| sequence-based reagent | Ccnb2 | ThermoFisher Scientific | Mm01171453_m1 |  |
| sequence-based reagent | Cdk1 | ThermoFisher Scientific | Mm00772472_m1 |  |
| sequence-based reagent | Cyr61 | ThermoFisher Scientific | Mm00487501_g1 |  |
| sequence-based reagent | Fgfr1 | ThermoFisher Scientific | Mm00438930_m1 |  |
| sequence-based reagent | HPRT | ThermoFisher Scientific | Hs02800695_m1 |  |
| sequence-based reagent | Icam1 | ThermoFisher Scientific | Mm00516023_m1 |  |
| sequence-based reagent | Kdr | ThermoFisher Scientific | Mm01222421_m1 |  |
| sequence-based reagent | Notch3 | ThermoFisher Scientific | Mm01345646_m1 |  |
| sequence-based reagent | Nr2f2 | ThermoFisher Scientific | Mm00772789_m1 |  |
| sequence-based reagent | Ptprc | ThermoFisher Scientific | Mm01293577_m1 |  |
| sequence-based reagent | Sema3c | ThermoFisher Scientific | Mm00443121_m1 |  |
| sequence-based reagent | Smad6 | ThermoFisher Scientific | Mm01171378_m1 |  |
| sequence-based reagent | SMAD6 | ThermoFisher Scientific | Hs00178579_m1 |  |
| sequence-based reagent | Smad7 | ThermoFisher Scientific | Mm00484742_m1 |  |
| sequence-based reagent | SMAD7 | ThermoFisher Scientific | Hs00998193_m1 |  |
| sequence-based reagent | Stat1 | ThermoFisher Scientific | Mm00439531_m1 |  |
| sequence-based reagent | Tgfbr2 | ThermoFisher Scientific | Mm00436977_m1 |  |
| sequence-based reagent | Ankrd33b | this paper | chr15:31359753-31360116 | F: AGTAAGAGTTAATTTAAGTGTGTTTG; R:CCCTTCCCTTAACCTCCTAATC |
| sequence-based reagent | Bmpr2 | this paper | chr1:59780665-59780973 | F: GTGGTTTATAATTTTAGTATTTGGG; R:TCTTTTATCTCTTAAATATATAATTTC |
| sequence-based reagent | Cd47 | this paper | chr16:49857758-49858224 | F: ATTTTTGTGGTAAAGGGGGAGTG; R:AACTACTAATACAACCCCACAAAC |
| sequence-based reagent | Cdkn2b | this paper | chr4:89306410-89306925 | F: TTATAATTTATTATTGGGTTTTGTGG; R:ACAAAACCAATCAAAAATAACTTCC |
| sequence-based reagent | Cyr61 | this paper | chr3:145648769-145648984 | F: TTTGGGGATATAGAGGAATGTAG; R:AAAACTTTACCATACATTAAATTAAAC |
| sequence-based reagent | Cyth3 | this paper | chr5:143630731-143631162 | F: TTGGGGTTGTTGGGAAATTAGAG; R:AAATCAATATAATTCCCACTAATTCC |
| sequence-based reagent | Dnmt3a | this paper | chr12:3892939-3893137 | F: AGGAGTAGTTGGTAGGTAAAGG; R:ATTAAAAATACTAAAACAACTATCAAC |
| sequence-based reagent | Ece1 | this paper | chr4:137864295-137864596 | F: TTATAGGGATTTTGGTAAAGTTGTG; R:CACAAAACAAATATCCTATAAATCTC |
| sequence-based reagent | Foxo3 | this paper | chr10:42273669-42273946 | F: AGTTAGAAGTTATAGAGTTTATAATG; R:TATTCCCTTTCAACCTCTTAAAAC |
| sequence-based reagent | Foxp1 | this paper | chr6:99103400-99103768 | F: TTATTTATTTAATGATGTTTTGAGATG; R:CAACTAAAATCTCTTTACAACTCC |
| sequence-based reagent | Hoxb2 | this paper | chr11:96353959-96354474 | F: AAAGTATTTATTGTATATTATTTTTATAG; R:ACCTAAACTTACAAACTCAAAACC |
| sequence-based reagent | Irf1 | this paper | chr11:53773525-53773975 | F: GGGTATGTGAGAAGTTTTAGGG; R:TTCCTCTTAATTTTACTCTTAATATC |
| sequence-based reagent | Itga1 | this paper | chr13:115099322-115099534 | F: TATTAAATAATAAGAAAGAGTAAGAGG; R:CCTCTTTAAAAATACCCATTCAATC |
| sequence-based reagent | Kitl | this paper | chr10:99957437-99957751 | F: GTTTTTAAAAGTAGTAAGTGTATGG; R:AATAACAAAACCTAATCCATTCTTC |
| sequence-based reagent | Nr2f2 | this paper | chr7:70357771-70358134 | F: AATAGGATTATGGTTTTGAGGTAG; R:CATACAACCTAACAACATCATAAAC |
| sequence-based reagent | Plxna1 | this paper | chr6:89356988-89357340 | F: TGTTTATATGGTTTGGGTAGTATAG; R:AAAACCAAACATATCAACATCTTCC |
| sequence-based reagent | Prickle2 | this paper | chr6:92443791-92444255 | F: GTTGAGAGATAGTTATTGGGTAG; R:TATTCTTTACCTCTAACAATCTAAC |
| sequence-based reagent | Sema3c | this paper | chr5:17576835-17577237 | F: GTTTAATTTAGATGAAATTTAAGGGG; R:TTCCCTTAAATATTAATTCCCAAATC |
| sequence-based reagent | Sema6a | this paper | chr18:47357782-47358200 | F: ATGTGTAAGGTAGGTAGATTATATG; R:ACTAAATTCTAAACAAACAACACAAC |
| sequence-based reagent | Smad6_1 | this paper | chr9:64007483-64007691 | F: TTTGGGATATTTGTTTTTTGTATG; R:CCCCCATCTAAATAACCCAC |
| sequence-based reagent | Smad6_2 | this paper | chr9:64001023-64001507 | F: AGTAGAATAATATAGTGATAGTTAGG; R:CCCAAATACCCTATCTACCCTC |
| sequence-based reagent | Smad7 | R&D Systems | chr18:75394281-75394619 | F: TGTGAAGGGTTGGGGTTAGTG; R:TCCGRACCATAAACTAATTTCTC |
| recombinant protein | Recombinant human BMP9 | R&D Systems | Cat#3209-BP-010 |  |
| recombinant protein | Recombinant human TGFB1 | R&D Systems | Cat#240-B-002/CF |  |
| commercial assay or kit | Arcturus PicoPure RNA Isolation Kit | ThermoFisher Scientific | Cat#KIT0204 |  |
| commercial assay or kit | ELISA Kit for Bone Morphogenetic Protein 6 (BMP6) | Cloud Clone | SEA646Mu |  |
| commercial assay or kit | Mouse BMP9 ELISA Kit | RayBiotech | ELM-BMP9 |  |
| commercial assay or kit | TGF-β1 Quantikine ELISA Kit | R&D systems | MB100B |  |
| commercial assay or kit | Click-iT EdU Flow Cytometry Assay Kit Alexa Fluor 647 | ThermoFisher Scientific | Cat#C10634 |  |
| commercial assay or kit | Click-iT Plus Alexa Fluor 555 Picolyl Azide Toolkit | ThermoFisher Scientific | Cat#C10642 |  |
| commercial assay or kit | MTT assay | Roche | Cat#000000011465007001 |  |
| commercial assay or kit | QIAamp DNA Micro Kit | Qiagen | Cat#56304 |  |
| commercial assay or kit | QuantiTect Reverse Transcription Kit | Qiagen | Cat#205313 |  |
| commercial assay or kit | RNeasy Mini Kit | Qiagen | Cat#205313 |  |
| commercial assay or kit | SMARTer Ultra Low RNA Kit for Illumina Sequencing | Clontech | Cat#634936 |  |
| chemical compound, drug | Collagenase I | Sigma | Cat#C9891-1g |  |
| chemical compound, drug | DMEM (Dulbecco's Modified Eagle's medium) | ThermoFisher Scientific | Cat#31885-049 |  |
| chemical compound, drug | DNaseI | Roche | Cat#11284932001 |  |
| chemical compound, drug | Dynabeads | ThermoFisher Scientific | Cat#11415D |  |
| chemical compound, drug | Endopan 3 HUVEC medium (+ supplement) | PAN Biotech | Cat#P04-0010B (P04-0010k) |  |
| chemical compound, drug | Mitomycin C | Sigma | Cat#M4287-2MG |  |
| chemical compound, drug | Neomycin/Geneticin | ThermoFisher Scientific | Cat#10131019 |  |
| chemical compound, drug | proteinase inhibitor mix | Serva Electrophoresis | Cat#39106.03 |  |
| chemical compound, drug | Puromycin | AppliChem | Cat#A2856,0025 |  |
| chemical compound, drug | RNase-free DNase Set | Qiagen | Cat#4444556 |  |
| chemical compound, drug | Sodium orthovanadate | Sigma | Cat#S6508-50g |  |
| chemical compound, drug | TaqMan Fast Advanced Mastermix | ThermoFisher Scientific | Cat#4444556 |  |
| software, algorithm | Bsseq, RRID:SCR_001072 | Hansen et al., 2012 | N/A |  |
| software, algorithm | BWA, RRID:SCR_010910 | Li and Durbin, 2009 | N/A |  |
| software, algorithm | DESeq2, RRID:SCR_015687 | Love et al., 2014 | N/A |  |
| software, algorithm | DIAMOnD algorithm | Ghiassian et al., 2015 | N/A |  |
| software, algorithm | Excel | Microsoft Office | N/A |  |
| software, algorithm | g:Profiler, RRID:SCR_006809 | Reimand et al., 2015 | N/A |  |
| software, algorithm | Galaxy, RRID:SCR_006281 | Afgan et al., 2016 | http://galaxyproject.org/ |  |
| software, algorithm | Gene Set Enrichment Analysis (GSEA) Software, RRID:SCR_003199 | Subramanian et al., 2005 | http://software.broadinstitute.org/gsea/index.jsp |  |
| software, algorithm | GenePattern, RRID:SCR_003201 | Broad Institute | http://software.broadinstitute.org/cancer/software/genepattern/ |  |
| software, algorithm | GraphPad Prism, RRID:SCR_002798 | GraphPad | https://www.graphpad.com/ |  |
| software, algorithm | GREAT tool, RRID:SCR_005807 | McLean et al., 2010 | http://great.stanford.edu/public/html/ |  |
| software, algorithm | HiSeq2000 platform | Illumina | N/A |  |
| software, algorithm | HOMER tools software package, RRID:SCR_010881 | Heinz et al., 2010 | N/A |  |
| software, algorithm | Image Analysis Fiji | Fiji | https://fiji.sc/ |  |
| software, algorithm | Integrative Genomics Viewer, RRID:SCR_011793 | Broad Institute | http://software.broadinstitute.org/software/igv/ |  |
| software, algorithm | Picard, RRID:SCR_006525 | N/A | http://picard.sourceforge.net/ |  |
| software, algorithm | Qiagens Ingenuity Pathway analysis, RRID:SCR_008653 | Ingenuity, IPA | http://www.ingenuity.com/ |  |
| software, algorithm | R statistical software v3.2.2 | R | https://cran.r-project.org/src/base/R-3/ |  |
| software, algorithm | SeqPrep, RRID:SCR_013004 | N/A | https://github.com/jstjohn/SeqPrep |  |
| software, algorithm | STAR, RRID:SCR_015899 | Dobin *et al*., 2013 | N/A |  |
| other | AGFA classic EOS developer | AGFA | N/A |  |
| other | Amersham Imager 600 | GE healthcare | N/A |  |
| other | BD Biosciences FACS Aria II | BD Biosciences | N/A |  |
| other | BD Biosciences FACS Canto II | BD Biosciences | N/A |  |
| other | Bioanalyzer | Agilent Technologies | Cat#G2940CA |  |
| other | HiSeq2000 Illumina machine | Illumina | N/A |  |
| other | MassARRAY | Agena Bioscience | N/A |  |
| other | Olympus Cell^R | Olympus | N/A |  |
| other | Olympus IX71 | Olympus | N/A |  |
| other | Qubit 3.0 Flourometer | Thermo Fisher | Cat#Q33216 |  |
| other | StepOnePlus Real-Time PCR System | Thermo Fisher | Cat#4376600 |  |
| other | Zeiss Cell Observer | Zeiss | N/A |  |
